# A complication of port-a-cath: disconnection and migration of central venous catheter to pulmonary artery. A case report.

**DOI:** 10.1186/2036-7902-4-S1-A24

**Published:** 2012-12-18

**Authors:** Maria Farré Pinilla, S Enriquez Bargalló, N Garcia Ruiz, P Espachs Biel, J Vilà Justribó, A Montero Matamala

**Affiliations:** 1 Department of Anesthesiology, Hospital Arnau de Vilanova, Lleida, Spain

## 

A 50 year old woman, with breast cancer undergoing chemotherapy. Login to removal because of the disconnection between the catheter and the subcutaneous port, diagnosed in routine check. In this case the X-ray showed the disconnection between the catheter and the subcutaneous port. And the consequent migration of the catheter, through cardiac cavities, into the pulmonary artery. Embolized catheter was removed by interventional radiology, under local anesthesia and intravenous sedation.The retrieval of the fragment was performed successfully using a snare catheter passed through the right femoral vein.

## Discussion

The central venous cannulation and placement of permanent vascular access is a common technique in cancer patients. This is an invasive procedure, non-therapeutic or curative in itself, which can lead to serious complications, even death.

The iconography of this case demonstrates a mechanical complication, potentially severe and rare placement of a port-a-cath. Embolized catheters can be removed by interventional radiology without significant adverse affects. The patient recovered without complications.
